# A Carbon Emission Calculation Model for Roadside Parking

**DOI:** 10.3390/ijerph18041906

**Published:** 2021-02-16

**Authors:** Wei Wang, Hongming Zhong, Yu Zeng, Yachao Liu, Jun Chen

**Affiliations:** School of Transportation, Southeast University, Nanjing 211189, China; wwang@seu.edu.cn (W.W.); 220193184@seu.edu.cn (Y.Z.); liuyachao@seu.edu.cn (Y.L.); chenjun@seu.edu.cn (J.C.)

**Keywords:** roadside parking, carbon emissions, model

## Abstract

With the sustained and rapid development of China’s national economy, the number of motor vehicles owned by families in cities is rapidly growing. Consequently, problems of traffic congestion and air pollution have also appeared in these cities. Roadside parking traffic has also become an important part of the transportation system in cities. However, there is no specific measurement model for carbon emissions caused by roadside parking in the proposed traffic carbon emission model. Therefore, we aim to establish a carbon emission measurement model for roadside parking. In this paper, we first study the characteristics of the deceleration and maneuvering of parking vehicles and the blocking impact on running vehicles in a typical roadside parking scenario. We then establish and fit models of the direct and indirect carbon emissions during roadside parking. Based on the carbon emission model, we propose a calculation method for roadside parking carbon emissions, including accounting and estimation methods. These models can be used to calculate the carbon emissions from roadside parking in a traffic carbon emissions system. We also hope that these models will help future research on the optimization of roadside parking facilities for energy saving and emission reduction.

## 1. Introduction

With the rapid development of the economy and motorization, the number of motor vehicles in China has increased rapidly, among which motor vehicles in cities account for a large part. Motor vehicles not only increase the convenience and accessibility of our travel but also cause pollution of the environment. Greenhouse gases, such as sulfur dioxide, nitrogen dioxide, and carbon dioxide, are considered to be the main causes of global climate change, which has attracted global attention [1]. Many experts have been paying attention to the relationship between carbon emissions and transportation. Wu pointed out that urban planning in the new period must take ecological civilization construction as the goal, take new development concepts led by innovation as the basic driving force, respect the basic laws of urban development and urbanization, and realize the multi-dimensional overall coordination of future urban development and the sustainable development of human urbanization [2,3,4]. Yu put forward a carbon emission measurement method for individual travel based on transportation big data and obtained the carbon emissions per kilometer or per time of passenger travel [5]. Yaacob published an overview of CO_2_ emissions from transportation and tried to update the methods used to calculate and analyze transportation carbon dioxide quickly and sub-optimally and presented different formulas used to measure CO_2_ emissions from transportation, including the methods of distance travel, fuel consumption, vehicle speed, vehicle type, and air-quality tool measurement [6].

Parking traffic, as part of urban traffic, plays an important role in residents’ daily travel and is gaining increasing public attention. Wang studied the time-varying effect of pricing on roadside parking characteristics and found that parking duration decreases as parking price increases, with the relationship presenting a growing elasticity [7]. Chien et al. investigated the feasibility of building a roadside parking management system mainly based on low-cost Bluetooth beacons, and their experiments showed that it suffices for correct detection of parking occupancy [8]. The carbon emission from roadside parking traffic is an important part of the traffic carbon emission system. However, at present, there are few studies on parking exhaust emissions, and even fewer on roadside parking exhaust emissions. Therefore, our research focuses on the carbon emissions caused by roadside parking, and we try to establish a measurement model of carbon emissions from roadside parking.

We define the research object of this article as follows:

1.  Roadside parking. Roadside parking refers to a parking mode in which a vehicle occupies the space set up for vehicles on the white line of the road. Roadside parking is mainly set up for the needs of travelers to temporarily park vehicles, which will occupy the road space and adversely affect the normal operation of traffic flow.
2.  Entering and exiting process of roadside parking. The entering and exiting process of roadside parking includes two processes, the process of a vehicle entering the parking space and the process of a vehicle exiting the parking space.
3.  Carbon emissions. There are many components in the exhaust emissions produced by cars stopping on the road. Among these carbon elements are included carbon dioxide (CO_2_), carbon monoxide (CO), and hydrocarbons (HC). In this study, the term carbon emissions refers to the emissions of CO_2_. In the automobile exhaust measurement experiment in this paper, the content of CO_2_ accounts for about 14% of the total exhaust gas, which is higher than the contents of CO and HC by 3–4 orders of magnitude. Therefore, CO_2_ is the main component of carbon emissions in automobile exhaust gas and, as a result, is the focus of this research. The experimental data are shown in Table A1 in the Appendix A.
4.  Direct carbon emissions from roadside parking. The direct carbon emissions from roadside parking are the carbon emissions from a vehicle in the process of the entering and exiting parking space. Direct carbon emissions have two main components: the basic carbon emissions from parking vehicles that are not affected by traffic flow and the carbon emissions from parking vehicles that are affected by traffic flow.
5.  Indirect carbon emissions from roadside parking. The indirect carbon emissions from roadside parking are caused by the delay of other vehicles on the road due to the impact of parking vehicles, which produces more carbon emissions than when the vehicles pass through the road freely. The amount of indirect carbon emissions is equal to the difference between the carbon emissions generated by traffic flow after setting roadside parking and the carbon emissions generated by traffic flow before setting roadside parking.

**Figure 1:**
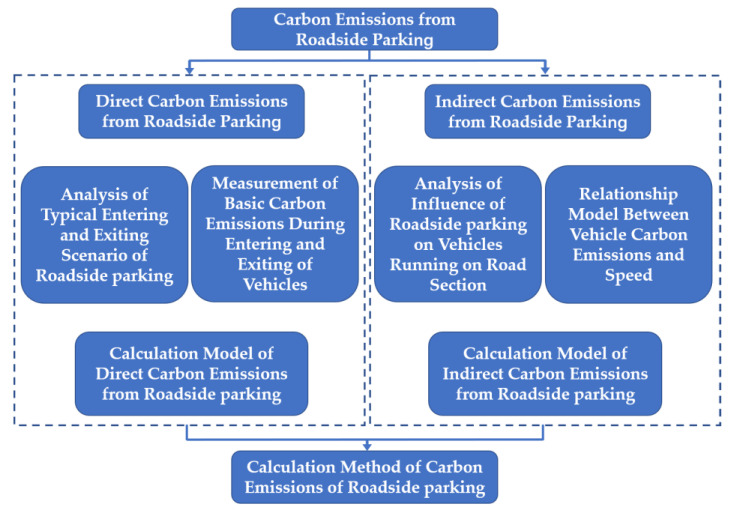
Research step diagram of the thesis.

The research ideas of this paper are as follows. First, we select typical parking scenarios of roadside parking by investigating the most common types of roadside parking. Then, we obtain the characteristic data and dynamic traffic characteristic data of the entering and exiting process under different parking scenarios. In addition, we perform qualitative and quantitative analysis of the characteristics of the entering and exiting process and their impact on traffic flow in different scenarios. Then, we design a scheme of carbon emission measurement experiment in typical roadside parking scenarios. By measuring the carbon emission of each scenario, we analyze the characteristics of direct carbon emissions from roadside parking and propose a calculation method for direct carbon emissions. Combined with previous research results, we analyze the indirect carbon emission characteristics of roadside parking and propose a calculation method. Finally, we combine the direct and indirect carbon emission models to establish the carbon emission calculation method of roadside parking, including the carbon emission accounting method and the estimation method of roadside parking. The research steps of this paper are shown in Figure 1.

The research methods of this paper mainly include a literature review, traffic investigation, and mathematical modeling. We hope to study and summarize the influencing factors of carbon emissions from roadside parking, the influence of roadside parking on dynamic traffic, and the applicable conditions and input requirements of the carbon emission calculation model by literature review. Through the method of traffic investigation, we want to obtain typical roadside parking settings and the characteristic data of the entering and exiting process in each scenario. Through mathematical modeling, we will study how to take the influence of roadside parking on traffic flow as the input condition or coefficient of the carbon emission calculation model and how to calibrate and optimize the traffic carbon emission calculation model.

Through this study, we aim to obtain a measurement model focusing on the carbon emissions generated during roadside parking. We believe that the results of this research will be helpful in the accounting of carbon emissions generated by roadside parking in the transportation carbon emission system and can be a supplement to the previous transportation carbon emission model.

## 2. Literature Review

Parking traffic, as an important part of the transportation system, has a corresponding influence on carbon emissions. Chester considered emissions by constructing the parking infrastructure when assessing the environmental impacts of vehicle travel [9]. Madiha reviewed the implementation cases of parking policies in Europe before 2019 and pointed out that these policies are aimed at promoting the reduction in traffic congestion and greenhouse gas emissions in Europe [10]. Zhao studied the urban traffic travel structure in low-carbon scenarios and pointed out that improving the urban traffic travel structure is an important way to improve traffic pollution and parking problems [11]. Wu studied the parking demand mechanism and established a graphical model based on microeconomics [12]. Roadside parking is an urgent issue to address in a fast-growing city of a developing country [13]. The carbon emissions from a roadside parking maneuver are an important part of the city traffic carbon emissions system. Therefore, it is of great significance to the research on the measurement methods of roadside parking emissions.

Vehicle emission data are mainly obtained through field tests and traffic simulations. The actual measurement methods mainly include the chassis dynamometer measurement system (CDMS), the tunnel inspection system, remote sensing detection (RSD), and vehicle exhaust detection. The CDMS uses a chassis dynamometer, an exhaust gas analyzer, a control computer, etc., to test the emission of an automobile in the preset driving cycle. CDMS technology is difficult and expensive, which cannot reflect the vehicle emissions on the actual road [14,15]. The tunnel inspection system calculates the concentration of pollutants produced when vehicles or train groups pass through a tunnel, by detecting parameters such as the concentration of pollutants at the entrance and exit of the tunnel and obtain the emission factor [16]. This method can truly reflect the actual level of vehicle emissions. However, it is difficult to operate because of the high requirements of experimental technology, and it is difficult to get different emission factors. Remote sensing detection (RSD) detects the concentration of the measured gas by using the change in infrared light and ultraviolet light intensity [17]. The method does not affect the normal running of automobiles, and the detection speed is fast. However, the detection points are required to be fixed, and there are high requirements for the location of the detection points and the surrounding environment. At the same time, this method can only measure the concentration of the detected gas but not the quality. The portable emission measurement system (PEMS) mainly includes a power supply, an exhaust sampling device, an exhaust analysis device, an exhaust calibration device, a fuel consumption meter, an airflow meter, engine data acquisition equipment, a velocimeter, and other parts. During the test, the PEMS is placed on the vehicle to be tested and can directly collect and measure the relevant parameters of vehicle operation, exhaust emission, engine condition, and so on so as to calculate the gas emission. The PEMS overcomes the shortcomings of the bench test, tunnel test, and RSD and can easily obtain emission data under different conditions, which has strong applicability. PEMS technology has been widely used in vehicle emission quantitative models [18,19].

In previous research, the commonly used traffic emission models were mainly the MOBILE (Motor Vehicle Emissions Factor Model) model, the COPERT (Computer Program to Calculate Emissions from Road Transport) model, and the MOVES (Motor Vehicle Emission Simulator) model. The MOBILE model can be used to calculate the comprehensive emission factor of a vehicle fleet. The calculation principle is to obtain the basic emission factor of the motor vehicle under standard operating conditions (Federal Test Procedure, FTP) based on emissions control technology of different vehicle models and then to modify the basic emission factor based on other factors under the actual operating conditions of the vehicle. Based on this, the single vehicle emission factor can be determined, and finally, the comprehensive emission factor can be obtained through the weighted average of the total emission factors of different vehicle models [20]. On the basis of the model, Gao compared and analyzed the influence factors of automobile exhaust emissions [21]. Wang studied the distribution of the road emission factors of diesel vehicles in Beijing and its surrounding areas [22]. Cheng made a comparative analysis of the MOBILE and COPERT models and pointed out that the COPERT model mainly uses reliable experimental technical data and traffic activity data to predict the pollutants emitted by motor vehicles. The COPERT model can calculate the amounts of pollutants emitted by a single vehicle or a fleet in one year [23]. The MOVES model is a new-generation vehicle emission model developed by the US Environmental Protection Agency based on the MOBILE and NONROAD series models. Its database is derived from a large amount of on-board carbon emissions test data and bench cumulative data. Because the MOVES model uses an open database management system, it has strong adaptability to different areas [24].

In summary, vehicle carbon emission measurement and road traffic carbon emission models are very rich, and each model has its own reasonable application scenarios. However, at present, the transportation carbon emission models, such as MOBILE, COPERT and MOVES, are mainly used for the carbon emissions from motor vehicles during operation, rather than focusing on the carbon emissions during parking. Although there is no specific carbon emission model for the roadside parking process at present, the study of vehicle carbon emission measurement and models provided by previous research can provide ideas and act as references for establishing a carbon emission model of roadside parking.

## 3. Materials and Methods

### 3.1. Typical Entering and Exiting Scenario of Roadside Parking

According to the characteristics and frequency of existing parking spaces in cities obtained from the investigation of four counties, combined with the influence of different types of parking spaces and parking modes on the carbon emissions, four of the most common and basic typical parking scenarios, A1, A2, B1, and B2, in cities were established, as shown in Table 1. For scenarios A1 and A2, in the actual parking process, forward and backward parking occur together in the same parking area, and the forward parking ratio is low. Therefore, in the following analysis, A1 and A2 are merged and denoted as A.

### 3.2. Analysis of Roadside Parking Entering and Exiting Process

**Figure 2:**
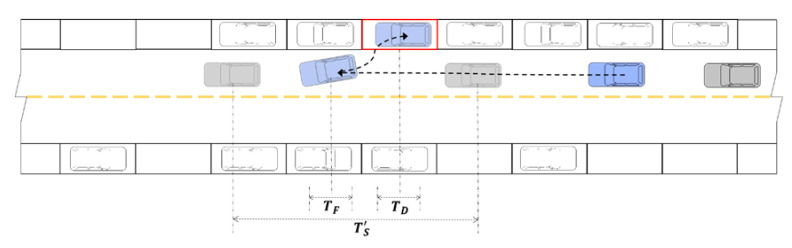
Schematic diagram of a vehicle entering a parking space.

**Figure 3:**
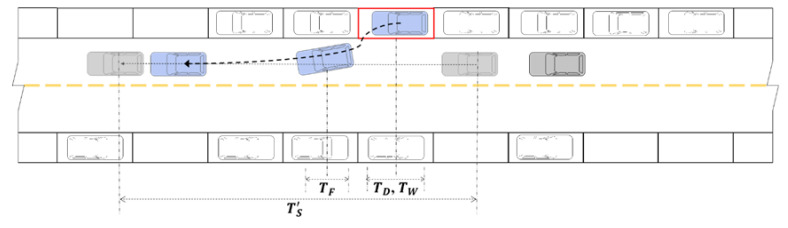
Schematic diagram of a vehicle exiting a parking space.

To study the direct and indirect carbon emissions characteristics of roadside parking, it is necessary to analyze the operating characteristics of parked vehicles and running vehicles. Complete roadside parking includes two processes, a vehicle entering and exiting the parking space, which are collectively called the entering and exiting process, as shown in Figure 2 and Figure 3, respectively.

As shown in Figure 2, the blue vehicle in the traffic flow is the parking vehicle, which is trying to park in the red target parking space. The gray vehicles are other vehicles moving with the traffic flow. When the blue vehicle passes by the target parking space and prepares to reverse into position, the driver observes a gray vehicle coming from behind. For the sake of safety, the blue vehicle may stop driving and let the gray vehicle pass by first, or the gray vehicle may stop moving forward and let the blue vehicle stop into the parking space before passing. In the whole process, this may occur several times until the blue vehicle stops in the parking space and the driver turns off the car.

As shown in Figure 3, the driver starts the blue vehicle, enters navigation settings and performs other operations within the vehicle, observes the traffic flow on the road, finds a suitable insertable gap (the time when the driver subjectively judges that he/she can safely leave the parking space), and then leaves the parking space and merges with the traffic flow. In this process, because the insertable gap is judged by the driver subjectively, the driver cannot guarantee that he/she can smoothly merge into the traffic flow. Therefore, when there are gray vehicles passing by before and after the blue vehicle, the above-mentioned scenario where the drivers stop driving and give way to each other may occur several times until the blue vehicle merges into the traffic flow.

Roadside parking under actual road conditions is split into two parts. One is the parking process, which the driver has to complete regardless of whether it is affected by the vehicles on the road section. The other is, after the vehicle is disturbed, it stops driving and gives way to other vehicles during parking. According to the above analysis, in the overall entering and exiting process, blue vehicles will be affected by traffic flow and stop several times. The time consumed in this process is called the stopping time related to traffic flow, which is denoted as $T_{F}$. In the process of parking the vehicle in the parking space, the driver may not turn off the vehicle immediately after the vehicle stops. In the process of leaving the parking space, after the vehicle is started, the driver may take actions such as setting navigation and interacting with a cell phone, followed by observing and merging into the traffic flow. In the above two processes, the vehicle is stationary, and this time consumed by the driver’s behavior is called the stopping time related to the driver, which is recorded as $T_{D}$. For the vehicle to merge into the traffic flow, the driver needs to spend a certain amount of time observing the traffic flow and judging the insertion opportunity. The time consumed in this process is called the stopping time related to finding an insertable gap, which is denoted as $T_{W}$. The total time consumed in the above process is called the total stopping time, which is recorded as $T_{S}$, where $T_{S}=T_{F}+T_{D}+T_{W}$.

After setting up roadside parking on the road, there will be two kinds of influences: friction and blocking. The friction effect of roadside parking on road traffic flow refers to the process by which parking occupies the space on both sides of the road, which narrows the passage space of the road in terms of the road geometry size and the driver’s visual and psychological influences, thus slowing down the speed of vehicles moving on the road. The blocking effect of roadside parking on the road traffic flow consists of two parts. First, vehicles running normally in traffic flow will slow down and give way due to the parking process of the parked vehicle. Second, when the parked vehicle leaves the parking space, the insertable gap is determined by the driver’s subjective judgment. Therefore, the driver cannot guarantee that the vehicle can smoothly merge into the traffic flow. This will cause the vehicles running on the road to be disturbed, resulting in stopping and blocking. In the above process, the delay caused to traffic flow by roadside parking is called the blocking time, which is denoted as $T_{S}^{\prime }$.

### 3.3. Measurement of Basic Carbon Emissions during Entering and Exiting of Vehicles

Pan used a portable emission measurement system (PEMS) to measure the emissions of gas pollutants from typical motor vehicles in Wuhan [25]. Hao measured the actual driving emissions from low-speed vehicles under the actual traffic conditions in rural areas using a PEMS [26]. In this study, the basic carbon emissions from typical parking vehicles in cities in common parking scenarios, A1, A2, B1, and B2, were measured by a vehicle-mounted exhaust gas analyzer SEMTECH-DS.

SEMTECH-DS has the precision of exhaust emission analysis in the laboratory and is used for the measurement of exhaust pollutants and particles from vehicle-mounted engines. The instrument uses the non-dispersive infrared (NDIR) method to analyze CO and CO_2_, uses the non-dispersive ultraviolet (NDUV) analysis method to collect relevant data of NO and NO_2_, and uses an electrochemical device to measure O_2_ in emissions. The measuring range, resolution, and accuracy of SEMTECH-DS are shown in Table 2.

During the experiment, besides the main engine of SEMTECH-DS, equipment such as a flowmeter and a collecting pipe are needed. After the collected tail gas reaches the flowmeter through the tail-gas-collecting pipe, the gas flow can be obtained. After the tail gas flows from the flowmeter to the host system of the tail gas detection device, various types of gas emission data can be obtained. Data are recorded once every second.

According to the data of vehicle ownership in four cities investigated in this paper, the Volkswagen LaVida (compact car, class A) with the highest ownership was selected as the experimental vehicle. The experimental vehicle was delivered in 2017, with a displacement of 1.6 L, a mass of 1245 kg, a maximum torque of 155 Nm, and a maximum power of 81 kw. The experimental oil was 92# gasoline of national V standard. The geometric dimensions of the parking space in each parking situation were 6 m × 2.5 m. The basic carbon emissions from typical roadside parking scenarios are shown in Table 3.

The experiment started at 10:00 a.m. The weather was good, the atmospheric temperature was about 25 °C, and it was breezy. Temperature and wind have little influence on tail gas collection.

### 3.4. Calculation Model of Direct Carbon Emissions from Roadside Parking

Combined with the in-depth analysis of the overall process of entering and exiting a parking spot on the road, the direct carbon emissions generated in the process of roadside parking were the sum of the basic carbon emissions and the carbon emissions generated in the three parking scenarios. Therefore, the following formula is given to describe the direct carbon emissions from roadside parking:

(1) $$E_{direct}=E_{B}+E_{D}+E_{W}+E_{F}=E_{B}+0.923*\left({\bar{T}}_{DW}+{\bar{T}}_{F}{}_{in}*{\bar{P}}_{in}+{\bar{T}}_{F}{}_{out}*{\bar{P}}_{out}\right),$$

where $E_{direct}$ represents the direct carbon emissions from roadside parking (g); $E_{B}$ represents the basic carbon emissions generated along the roadway segment facilitating roadside parking (g), which can be obtained by referring to Table 3; $E_{D}$ represents the carbon emissions generated by driver-related activities during roadside parking (g); $E_{W}$ represents the carbon emissions generated in the process of searching for the neutral gear in the process of roadside parking (g); $E_{F}$ represents the carbon emissions caused by the dynamic traffic impact during roadside parking (g); ${\bar{T}}_{DW}$ is the average stopping time related to the driver’s search for the neutral gear in the process of roadside parking (s); ${\bar{T}}_{Fin}$ is the relative average stopping time of the traffic flow while parking in a parking space in the roadside parking process (s); ${\bar{P}}_{in}$ is the relative average stopping probability of the traffic flow while parking in a parking space in the roadside parking process (dimensionless); ${\bar{T}}_{Fout}$ is the relative average stopping time of the traffic flow while leaving the parking space during the roadside parking process (s); ${\bar{P}}_{out}$ is the relative average stopping probability of the traffic flow while leaving the parking space in the roadside parking process (dimensionless); and 0.923 is the CO_2_ emissions per second during the parking process when the running speed of the vehicle is 0 km/h (g).

### 3.5. Influence of Roadside Parking on Vehicles Running on the Road Section

#### 3.5.1. Friction Effect of Roadside Parking on Traffic Flow on the Road Section

Different types of roadside parking space on the road section occupy different road spaces and have different friction effects on the traffic flow on the road. Mei introduced the spatial obstacle ratio to describe the friction effect of roadside parking on road traffic flow, the so-called spatial obstacle ratio $R_{b}$ [27,28]. This refers to the ratio of the road width b occupied by a roadside parking strip to the total road width B, which is expressed as follows:

(2) $$R_{b}=b/B$$

where $R_{b}$ is the spatial obstacle ratio, B is the pavement width of the road with roadside parking (m; note: this excludes sidewalks and green belts; if it is set on one side, it is the width of the road on one side of the parking belt; if it is set on both sides, it is the total width of the road), and b is the width of the road occupied by the roadside parking (m; excluding sidewalks and green belts), which is set based on the geometric size of the standard parking space. Generally, b is 2.5 or 5 m when one or both sides of the road are provided with parallel roadside parking; when the vertical parking spaces were set on one or both sides of the road, b was 6 or 12 m.

**Figure 4:**
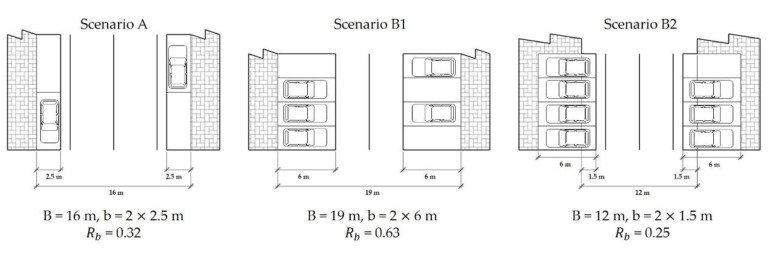
Geometric parameters of road sections in each scenario.

The geometric parameters of each scenario in this paper were obtained through investigation, as shown in Figure 4. In addition, their spatial obstacle ratios were calculated, as shown in Table 4.

#### 3.5.2. Blocking Effect of Roadside Parking on Traffic Flow

Different types of roadside parking garages and parking modes were adopted in each scenario, and the influence time of blocking on adjacent lanes was also different. Moreover, the higher the frequency of moving in and out of the parking space is, the greater the influence on the traffic flow blockage becomes. The frequency of moving in and out of a parking space is proportional to the turnoff of the roadside parking. The higher the turnover ratio is, the higher the frequency is. The data of the blocking impact time obtained from the survey were statistically analyzed to obtain the average blocking impact time of roadside parking in three typical scenarios, as shown in Table 5.

Table 5 shows that the processes of entering and leaving a parking spot during roadside parking have different congestion effects on the formation of road traffic flow. Therefore, when calculating the total influence time $T^{\prime }$ in the interval, we should calculate and sum the two processes separately, that is, the mode factor and frequency factor of the parking and leaving vehicles should be combined, as follows:

(3) $$T^{\prime }=n_{in}\times T_{S}^{\prime }{}_{in}+n_{out}\times T_{S}^{\prime }{}_{out}$$

where $n_{in}$ is the number of parked vehicles in the statistical interval (veh), $T_{S}^{\prime }{}_{in}$ is the average blocking time during the parking process (s), $n_{out}$ is the number of vehicles leaving in a statistical interval (veh), and $T_{S}^{\prime }{}_{out}$ is average blocking time during the departure process (s).

Mei once introduced the time barrier rate $R_{t}$ to describe the blocking effect of roadside parking on the road traffic flow [27,28], i.e., the proportion of the total influence time $T^{\prime }$ in the statistical interval to the statistical interval time $T_{interval}$, expressed as follows:

(4) $$R_{t}=T^{\prime }/T_{interval}$$

### 3.6. Traffic Flow Model in a Typical Roadside Parking Scenario

According to the investigation and analysis of the dynamic and static traffic interaction characteristics in typical scenarios of roadside parking in cities, the load intensity of the road sections rarely exceeds 0.8. Thus, in the research scenario of roadside parking in cities, there is basically no scenario in which the road flow is close to its actual capacity. Therefore, it is most appropriate to construct a basic model to characterize the influence of roadside parking on the road traffic flow based on the BPR (Bureau of Public Road) model of the Bureau of Public Road of USA. The basic formula is as follows:

(5) $$v\left(q_{i},v_{0},c_{i}\right)=\frac{v_{0}}{\prod_{i}\left[1+\alpha_{i}{\left(q_{i}/c_{i}\right)}^{\beta_{i}}\right]}$$

where *i* is the traffic flow type, $v\left(q\right)$ is the speed of vehicles running on the road section when the flow rate is q (km/h), $v_{0}$ is the running speed (km/h; that is, the running speed of vehicles on the road section when the flow rate is 0), c is the actual traffic capacity of the road section (veh/h), and α and β are model parameters.

The above model describes the influence of the road traffic load on the running speed. According to the BPR model, it is assumed that under specific road flow conditions, when different types of roadside parking are set on the roadside, the actual traffic capacity $c_{i}$ of the road and the speed decrease by various degrees. When considering the influence of roadside parking on the traffic flow, we should comprehensively consider the reduction caused by friction and blocking effects.

The friction effect caused by the roadside parking zone is mainly reflected by the spatial obstacle ratio $R_{b}$. Based on the previous analysis, different types of roadside parking have different friction effects on the road traffic flow due to their different spatial obstacle ratios. The degree of friction influence increases with an increase in the spatial obstacle ratio, which leads to a decrease in the running speed of the dynamic traffic flow and has a negative linear relationship with the spatial obstacle ratio $R_{b}$. Therefore, using the spatial obstacle ratio $R_{b}$ to linearly reduce Equation (5), we obtain the following:

(6) $$v\left(q_{i},v_{0},c_{i}\right)=\left(1-k_{1}R_{b}\right)\frac{v_{0}}{\prod_{i}\left[1+\alpha_{i}{\left(q_{i}/c_{i}\right)}^{\beta_{i}}\right]}$$

In addition, the blocking effect caused by the roadside parking area is mainly reflected by the time barrier ratio $R_{t}$. Based on the previous analysis, different types of roadside parking have different blocking effects on the road traffic flow due to their different time barrier ratios. With the increase in the time barrier rate, the time of the blocking effect increases, which leads to a decrease in the running speed of the dynamic traffic flow and has a quadratic function relationship with the time barrier ratio $R_{t}$ negatively. Therefore, Equation (6) is reduced twice by using the spatial obstacle ratio $\;R_{t}$, and the following is obtained:

(7) $$v\left(q_{i},v_{0},c_{i}\right)=\left(1-k_{1}R_{b}-k_{2}R_{t}^{2}\right)\frac{v_{0}}{\prod_{i}\left[1+\alpha_{i}{\left(q_{i}/c_{i}\right)}^{\beta_{i}}\right]}$$

Considering the motor vehicle and non-motor vehicle flows in the model, we obtain the following:

(8) $$v\left(q_{i},v_{0},c_{i}\right)=\left(1-k_{1}R_{b}-k_{2}R_{t}^{2}\right)\frac{v_{0}}{\left[1+\alpha_{m}{\left(q_{m}/c_{m}\right)}^{\beta_{m}}\right]\cdot \left[1+\alpha_{n}{\left(q_{n}/c_{n}\right)}^{\beta_{n}}\right]}$$

where $q_{m}/c_{m}$ is the saturation of forward motor vehicle flow, $q_{n}/c_{n}$ is the saturation of forward non-motor vehicle flow, and $k_{1}$ and $k_{2}$ are model parameters.

### 3.7. Relationship Model between Vehicle Carbon Emissions and Speed

There is a linear relationship between the carbon emissions and fuel consumption level of a vehicle during driving, which can be expressed by the following formula [29]:

(9) E=m×ρ×N×F×I

where E represents the carbon emissions (mg), m is the fuel consumption (L), ρ is the fuel density (kg/L), N is the default net heating value of the fuel (MJ/kg), F is the emission factor based on the net heating value (mg/MJ), and I is characteristic factor (dimensionless).

At the same time, the relationship between the fuel consumption level and the vehicle running speed is a quadratic function [29], i.e.,

(10) $$m=\alpha v^{2}-\beta v+\gamma$$

where v is the running speed of the vehicle (km/h) and α, β, and γ are model parameters.

Therefore,

(11) $$E=\left(\alpha v^{2}-\beta v+\gamma \right)\times \rho \times N\times F\times I$$

Because the research content of this paper is not directly related to the fuel type, the simplified formula in the study is as follows:

(12) $$E=\alpha v^{2}-\beta v+\gamma$$

### 3.8. Calculation Model of Indirect Carbon Emissions from Roadside Parking

The indirect carbon emissions from roadside parking are the extra carbon emissions from vehicles operating on the road due to the interference of roadside parking, i.e., the difference between the carbon emissions generated by road traffic flow after and before setting up roadside parking. Based on the difference between the BPR function in three typical scenarios and the BPR function of the road section without roadside parking, the speed reduction caused by roadside parking is obtained, and the calculation model of the indirect carbon emissions from roadside parking can be obtained by substituting it into the expression relating carbon emissions and speed.

Historical research shows that when there is no roadside parking on the road section, the traffic flow state of the two-way, two-lane road can be expressed by the following formula [27]:

(13) $$v=\frac{v_{0}}{\left[1+0.694{\left(q_{m}/c_{m}\right)}^{1.678}\right]\cdot \left[1+0.703{\left(q_{n}/c_{n}\right)}^{1.778}\right]}$$

Therefore, the calculation formula of the indirect carbon emission model is as follows:

(14) $$E_{indirect}=\alpha {\left(v_{1}-v_{2}\right)}^{2}-\beta \left(v_{1}-v_{2}\right)+\gamma$$

where $v_{1}$ is calculated using Equation (8) and $v_{2}$ is calculated using Equation (13).

## 4. Results and Discussion

### 4.1. Calibration of Direct Carbon Emission Model for Roadside Parking

The data obtained from the investigation were processed, and the  V/C ratio of the inbound and outbound traffic flows was matched to obtain the data format shown in Table 6. The direct carbon emissions value $E_{direct}$ under the corresponding V/C ratio was calculated using Equation (1).

**Figure 5:**
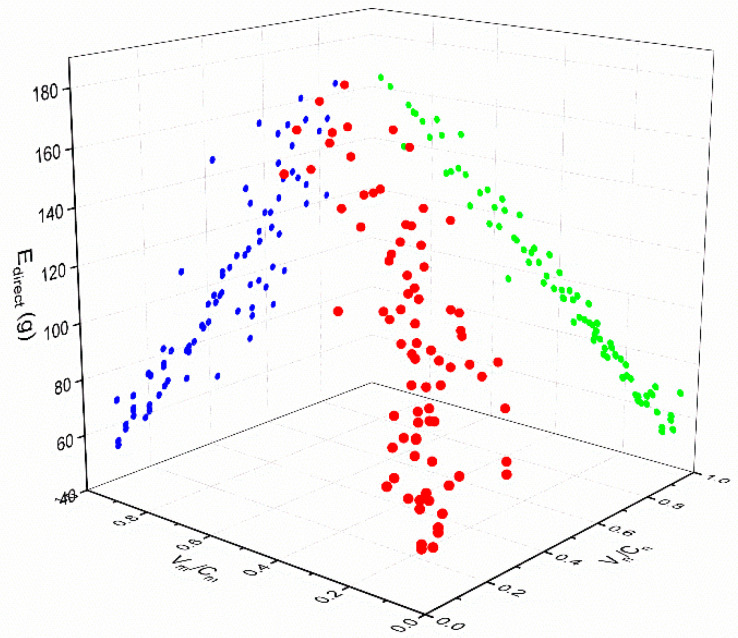
Relationship between direct carbon emissions and road saturation in scenario A.

**Figure 6:**
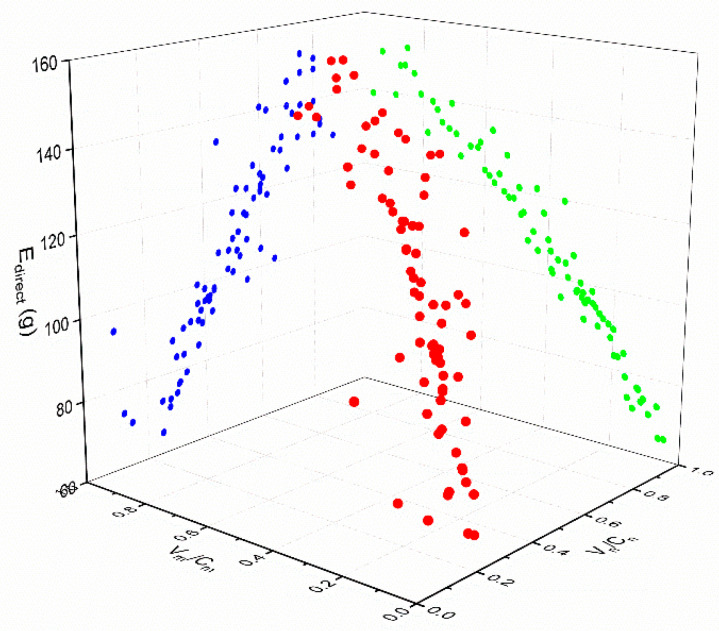
Relationship between direct carbon emissions and road saturation in scenario B1.

**Figure 7:**
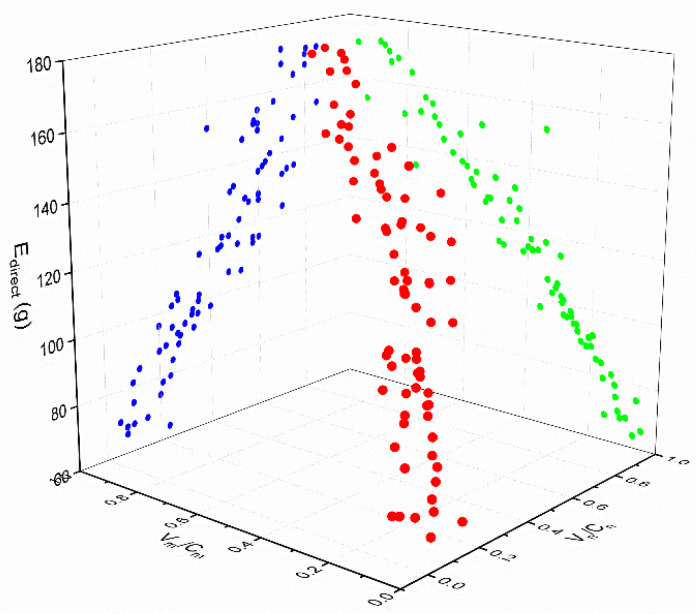
Relationship between direct carbon emissions and road saturation in scenario B2.

First, according to the data in the above table, a scatter diagram was drawn to show the relationships between $E_{direct}$ and $V_{m}/C_{m}$ and between $E_{direct}$ and $V_{n}/C_{n}$ in each scenario, as shown in Figure 5, Figure 6 and Figure 7 (in the spatial rectangular coordinate system, the *x* axis represents $V_{n}/C_{n}$, the y axis represents $V_{m}/C_{m}$, and the z axis represents $\;E_{direct}$). The red points are drawn according to the coordinates. The blue points and green points are generated by projecting the red points onto the *XOZ* plane and the *YOZ* plane, respectively.). The scatter plot can be used to determine the trend line, so it can be judged that the direct carbon emissions in each scenario were linearly and positively correlated with the saturation of motor vehicles and non-motor vehicles. Therefore, we suppose that

(15) $$E_{direct}=\alpha \cdot V_{m}/C_{m}+\beta \cdot V_{n}/C_{n}+\varepsilon$$

where α, β, and ε are the parameters to be calibrated.

In this study, OriginPro2020 software (OriginLab, Northampton, MA, USA) was used to fit the model, and the multiple linear regression module was used to fit the direct carbon emission model for roadside parking. The fitting results of the models for scenarios A, B1, and B2 are shown as Equations (16)–(18).

- Scenario A:
  (16) $$E_{direct}=109.505\cdot V_{m}/C_{m}+14.875\cdot V_{n}/C_{n}+45.7$$
- Scenario B1:
  (17) $$E_{direct}=76.403\cdot V_{m}/C_{m}+23.685\cdot V_{n}/C_{n}+63.494$$
- Scenario B2:
  (18) $$E_{direct}=94.554\cdot V_{m}/C_{m}+29.318\cdot V_{n}/C_{n}+62.849$$

### 4.2. Fitting of the BPR Model in a Typical Roadside Parking Scenario

In the past, the smooth speed $v_{0}$, traffic load intensity $q_{i}/c_{i}$, and speed $\;v\left(q_{i},v_{0},c_{i}\right)$ of running vehicles in different roadside parking scenarios were obtained through investigation (Table A2, Table A3 and Table A4 in the Appendix A). The BPR models in scenarios A, B1, and B2 were calibrated using the above data, and the influence of roadside parking on the speed of traffic flow on the road was analyzed.

Using the nonlinear curve fitting module of OriginPro2020, Equation (8) was added to the function library of the software in the form of a user-defined function, and the BPR function was recalibrated using the actual data, as shown in Table 7.

### 4.3. Calibration of the Relationship Model between Vehicle Carbon Emissions and Speed

To calibrate the relationship between the carbon emission rate and the speeds of motor vehicles operating on the road sections, a vehicle-mounted exhaust gas analyzer SEMTECH-DS was used to measure the carbon emissions. Because the road section where roadside parking was set was a branched road or a secondary trunk road, the traveling speed was typically about 40 km/h, the design speed was between 20 and 50 km/h, and the vehicle speed was between 0 and 30 km/h when the vehicles passed slowly under the influence of roadside parking. Therefore, the experimenter drove a Volkswagen LaVida to run repeatedly between 0 and 50 km/h and recorded the carbon emission data using the exhaust gas detector (see data in Table A5 in the Appendix A).

**Figure 8:**
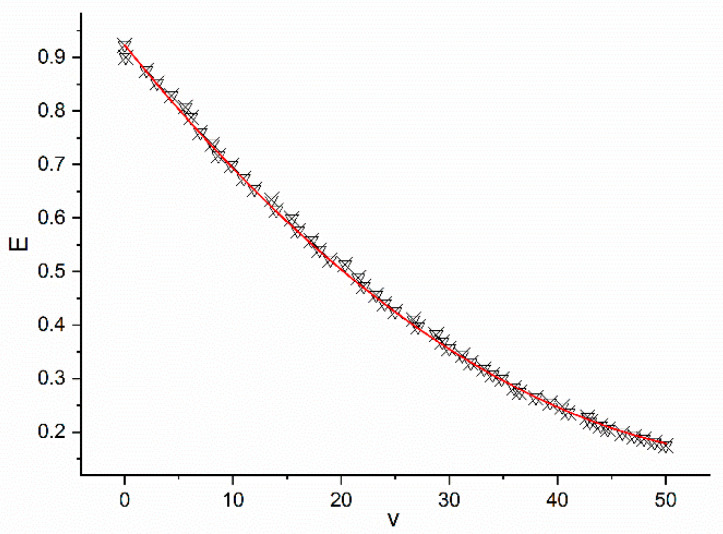
Relationship between carbon emissions and velocity.

Using the nonlinear curve fitting module of OriginPro2020, Equation (12) was calibrated, and the relationship between carbon emissions and velocity is shown in Figure 8 (the horizontal axis represents velocity (v), the vertical axis represents carbon emissions (E), and the red line is the fitting line of the data.).

The fitted coefficients were α=0.0002, β=−0.0245, and γ=0.9232, and the relationship between the carbon emissions and velocity was as follows:

(22) $$E=0.0002v^{2}-0.025v+0.923$$

The correlation coefficient of the calibration results was R^2^ = 0.979, and the statistical value of F was 26886.608, which met the significance level of 0.05.

### 4.4. Calibration of the Indirect Carbon Emission Calculation Model for Roadside Parking

The BPR functions (Equations (19)–(21)) in three typical scenarios were different from the BPR function (Equation (13)) of the road section without roadside parking, and the reduced value of the vehicle flow speed caused by roadside parking was obtained, which was substituted into the fitted expression relating carbon emissions and speed (Equation (22)), as presented in the previous section. The indirect carbon emission models of scenarios A, B1, and B2 of roadside parking were then obtained. Take scenario A as an example, as shown below.

Indirect carbon emission model of scenario A:

(23) $$E_{indirect}=0.0002{\left(\frac{\left(1-2.961R_{b}-0.252R_{t}^{2}\right)\cdot v_{0}}{\left[1+105.011{\left(q_{m}/c_{m}\right)}^{6.168}\right]\cdot \left[1-0.945{\left(q_{n}/c_{n}\right)}^{-0.002}\right]}-\frac{v_{0}}{\left[1+0.694{\left(q_{m}/c_{m}\right)}^{1.678}\right]\cdot \left[1+0.703{\left(q_{n}/c_{n}\right)}^{1.778}\right]}\right)}^{2}\;\;\;\;\;\;\;\;\;\;\;\;\;-\frac{0.025\left(1-2.961R_{b}-0.252R_{t}^{2}\right)\cdot v_{0}}{\left[1+105.011{\left(q_{m}/c_{m}\right)}^{6.168}\right]\cdot \left[1-0.945{\left(q_{n}/c_{n}\right)}^{-0.002}\right]}+\frac{0.025v_{0}}{\left[1+0.694{\left(q_{m}/c_{m}\right)}^{1.678}\right]\cdot \left[1+0.703{\left(q_{n}/c_{n}\right)}^{1.778}\right]}+0.923$$

The meaning of the indirect carbon emission model is further explained. The model expresses the indirect carbon emission rates of vehicles operating in traffic flow under specific traffic conditions with motor vehicles and non-motor vehicles on a road with roadside parking when the smooth speed and capacity of the road are known. The unit of the indirect carbon emission rate is g/(s·veh).

### 4.5. Calculation Method of Carbon Emissions from Roadside Parking

#### 4.5.1. Accounting Method of Total Carbon Emissions from Roadside Parking

According to the above research, if a certain type of roadside parking is set up on a road section, the carbon emissions will be composed of direct carbon emissions and indirect carbon emissions from roadside parking. To accurately calculate the total carbon emissions from roadside parking in current cities, it is necessary to obtain the motor vehicle saturation $V_{m}/C_{m}$, non-motor vehicle saturation $V_{n}/C_{n}$, smooth speed $\;v_{0}$, spatial obstacle ratio $R_{b}$, time obstacle ratio $R_{t}$, and daily cumulative parking time $N_{onsrteet}^{i}$ of all the road sections through investigation and list the conditions of the roadside parking in all road sections. At the same time, we should obtain the traffic volume $Q_{m}^{i}$ of the road section and the time $T_{i}$ required for motor vehicles to pass through the road section, calculate their respective carbon emissions, and then summarize the results to obtain the overall carbon emissions from roadside parking in the current cities.

Taking one day as an example, the carbon emissions from roadside parking in cities can be calculated by the following formula:

(24) $$E_{onstreet}=\underset{i}{\sum }E_{onstreet}^{i}=\underset{i}{\sum }E_{direct}\times N_{onsrteet}^{i}+E_{indirect}\times Q_{m}^{i}\times T_{i}$$

where $E_{onstreet}$ represents the total carbon emissions generated by roadside parking in cities in one day (g/d); $E_{onstreet}^{i}$ represents the carbon emissions from roadside parking in section i of the city (g/d); $E_{direct}$ represents the direct carbon emissions from one roadside parking event (g/time), which can be calculated using Equations (16)–(18); $N_{onsrteet}^{i}$ is the cumulative number of parking events in the roadside parking zone of section *i* of the city (time/d); $E_{indirect}$ is the carbon emission rate of the vehicles driving on the roadside parking section (g/(s·veh)), which can be calculated by Equations (19)–(22); $Q_{m}^{i}$ is the traffic volume of section i of the city (veh/d); and $T_{i}$ is the average time required for vehicles to pass through section *i* (s).

Therefore, the carbon emissions from all roadside parking lots in cities were $7\cdot E_{onstreet}$ in one week, $30\cdot E_{onstreet}$ in one month, and $365\cdot E_{onstreet}$ in one year.

#### 4.5.2. Estimation Method of Total Carbon Emissions from Roadside Parking

Based on the presented scenario of roadside parking in cities, the overall carbon emissions from roadside parking were estimated. First, reasonable default values for the parameters of the three kinds of roadside parking were set, as summarized in Table 8.

Therefore, the direct carbon emissions from one roadside parking event were as follows: 65.413 g/time for scenario A, 77.323 g/time for scenario B1, and 79.964 g/time for scenario B2, and the average of the three was 74.429 g/time (by Equations (16)–(18)). Similarly, the average level of the indirect carbon emissions was 1.099 g/(s·veh) (by Equations (19)–(22)).

Taking one day as an example, the total carbon emissions from roadside parking in cities are calculated as follows:

1. Calculate the total direct carbon emissions.

The direct carbon emissions are calculated as follows:

(25) $$E_{Sdirect}=74.429\times n_{onstreet}\times r_{onstreet}$$

where $E_{Sdirect}$ is the total amount of direct carbon emissions generated by one-day roadside parking in cities (g/d), $n_{onstreet}$ is the number of roadside parking berths in cities, and $r_{onstreet}$ is the daily average accumulated parking time of a single roadside parking berth in cities (time/d).

2. Calculate the total indirect carbon emissions.

From the investigation of cities, the average speed of the roadside parking section was about 15 km/h, and the total length S (km) of roadside parking roads and the total traffic volume Q (veh) on the roadside parking sections in cities were obtained. The indirect carbon emissions were as follows:

(26) $$E_{Sindirect}=1.099\times Q\times \frac{S}{15}\times 3600$$

where $E_{Sindirect}$ represents the total indirect carbon emissions generated by one day of roadside parking in cities (g/d).

3. Calculate the total carbon emissions.

The total carbon emissions generated by one day of roadside parking in cities is as follows:

(27) $$E_{onstreet}=E_{Sdirect}+E_{Sindirect}$$

Therefore, the carbon emissions from all roadside parking lots in cities are $7\cdot E_{onstreet}$ in one week, $30\cdot E_{onstreet}$ in one month, and $365\cdot E_{onstreet}$ in one year.

## 5. Conclusions

The starting point of this paper was to supplement the previous research on the traffic carbon emission model, and the carbon emission characteristics of roadside parking in typical parking scenarios were studied. Based on the field investigation and the analysis of the influence factors of roadside parking carbon emissions, four typical parking scenarios of roadside parking were summarized and selected. Through the analysis of the interaction characteristics between roadside parking and dynamic traffic, it was further concluded that there were three major scenarios, A, B1, and B2, that had significant impacts on the carbon emissions.

We designed and implemented roadside parking carbon emission measurement experiments, and we obtained the basic carbon emissions during roadside parking in three main typical scenarios. Through the analysis of the parking process of roadside parking and the analysis of the characteristics of vehicle entering and exiting, we proposed a direct carbon emission model for roadside parking and calibrated it. We calibrated the BPR function of road traffic flow in a typical parking scenario through the analysis of the process of roadside parking and the friction and blocking effect of roadside parking on the traffic flow. We also fitted the model relating the vehicle carbon emissions and speed. We then obtained the indirect carbon emission model of roadside parking. Finally, we further proposed the calculation method of roadside parking carbon emissions. The purpose of the current study was to obtain a calculation model focusing on the carbon emissions generated during roadside parking. The calculation model of this research can be used to calculate the carbon emissions from roadside parking, and we hope it will be helpful for other researchers studying automotive emissions.

As the current measurement methods for transportation carbon emissions are not yet mature, there are few theories and research results that can be used for reference, and the available cases and available data are relatively limited. Although some results have been made in this paper, there are still some shortcomings and defects. In the basic carbon emission measurement experiment, we only selected the automotive type with the highest number to test. If conditions permit, we should measure the basic carbon emissions from more automotive types in the future study. In further research, we should analyze more roadside parking scenarios in practice and consider the parking time, the size of the parking space, the distance between parking spaces, space availability, occupancy, and the influence of different environmental conditions. In our research, we studied carbon emissions under a constant environmental condition, and we regarded the environmental condition as a constant. However, in practice, environmental conditions should be a variable, and carbon emissions are different under different environmental conditions. Although it is very difficult to study the carbon emissions caused by roadside parking under all environmental conditions, we can select several typical environmental conditions for research in the future. The interaction mechanism between roadside parking vehicles and dynamic traffic of a road section also requires further study.

## Figures and Tables

**Table 1:** Typical parking scenarios of roadside parking.

Scenario		Isolation between Machine and Non-machine	Design Type of Parking Space	Entering and Exiting Mode of Vehicle	Sketch Map
A	A1	No	Parallel	Forward-park inForward-drive out	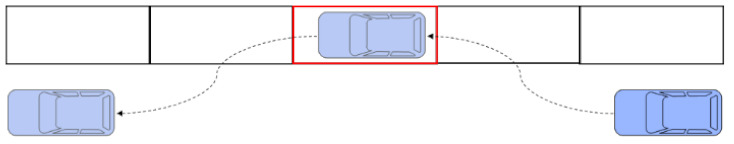
	A2	No	Parallel	Back-park inForward-drive out	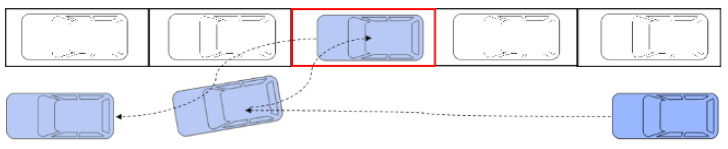
B1		No	Vertical on roadside	Back-park inForward-drive out	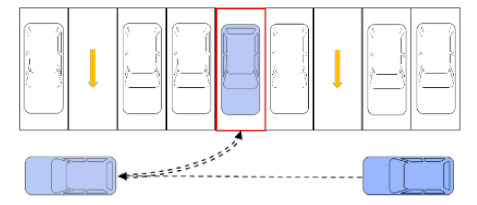
B2		No	Vertical on sidewalk	Forward-park inBack-drive out	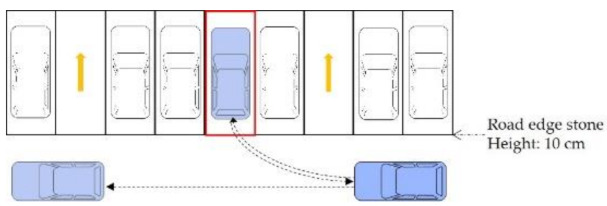

**Table 2:** Measuring range, resolution, and accuracy of SEMTECH-DS.

Gas	Measuring Range	Resolution	Accuracy
CO	0–8%	10 ppm	±3%
CO_2_	0–20%	0.01%	±3%
NO	0–2500 ppm	1 ppm	±3%
NO_2_	0–500 ppm	1 ppm	±3%
O_2_	0–25%	0.1%	±1%

**Table 3:** Basic carbon emissions from typical roadside parking modes.

Scenario		Parking Mode		Average Time (s)	Carbon Emissions (g)	Total Carbon Emissions (g)
A	A1	Parallel	Forward-park in	11.55	8.457	14.788
			Forward-drive out	8.50	6.331	
	A2	Parallel	Back-park in	33.70	26.117	34.278
			Forward-drive out	10.35	8.161	
B1		Vertical on roadside	Back-park in	35.89	31.792	39.766
			Forward-drive out	12.39	7.974	
B2		Vertical on sidewalk	Forward-park in	27.75	23.393	40.711
			Back-drive out	21.58	17.318	

**Table 4:** Spatial obstacle ratio of typical roadside parking scenarios.

Scenario	Geometric Parameters of the Road Section	Spatial Obstacle Ratio
A	Road width 16 m, two-way, two-lane, parallel parking spaces set on both sides	0.32
B1	Road width 19 m, two-way, two-lane, vertical parking spaces set on both sides	0.63
B2	Road width 12 m, two-way, two-lane, vertical parking spaces set on both sides of the road and sidewalk	0.25

**Table 5:** Mean duration of blocking effect of typical roadside parking scenarios.

Typical Scenario		Mean Duration of Blocking Effect (s)
A	Parking in	9.90
	Parking out	5.28
B1	Back-park in	12.14
	Forward-drive out	6.97
B2	Forward-park in	8.09
	Back-drive out	13.25

**Table 6:** Summary of direct carbon emission parameters during parking in scenario A (example).

$V_{m}/C_{m}$	$V_{n}/C_{n}$	${\bar{T}}_{DW}$	${\bar{T}}_{F}{}_{in}$	${\bar{T}}_{F}{}_{out}$	${\bar{P}}_{F}{}_{in}$	${\bar{P}}_{F}{}_{out}$	$E_{direct}$
0.046	0.323	39.744	0	0	0	0	68.039
0.538	0.65	61.744	20.631	9.92	0.75	1	111.783
…	…						…

$V_{m}/C_{m}$ is the saturation of the motor vehicle flow, and $V_{n}/C_{n}$ is the saturation of the non-motor vehicle flow. Please note that the ellipsis indicates that only part of the data is shown in the table. It means the same in other tables.

**Table 7:** Summary of the BPR-modified model for each scenario.

Typical Scenario	BPR-Modified Model	
A	$v=\frac{\left(1-2.961R_{b}-0.252R_{t}^{2}\right)\cdot v_{0}}{\left[1+105.011{\left(q_{m}/c_{m}\right)}^{6.168}\right]\cdot \left[1-0.945{\left(q_{n}/c_{n}\right)}^{-0.002}\right]}$	(19)
B1	$v=\frac{\left(1-0.165R_{b}-1.416R_{t}^{2}\right)\cdot v_{0}}{\left[1+167.343{\left(q_{m}/c_{m}\right)}^{5.211}\right]\cdot \left[1-6.229{\left(q_{n}/c_{n}\right)}^{18.743}\right]}$	(20)
B2	$v=\frac{\left(1-0.011R_{b}-0.016R_{t}^{2}\right)\cdot v_{0}}{\left[1+123.735{\left(q_{m}/c_{m}\right)}^{6.230}\right]\cdot \left[1-0.123{\left(q_{n}/c_{n}\right)}^{0.157}\right]}$	(21)

**Table 8:** Default values of each parameter.

Parameter	Default Value	Explanation
$v_{0}$	40 km/h	Road sections with roadside parking in cities should be secondary trunk roads and branch roads. According to relevant design specifications, the design speeds of secondary trunk roads are 50, 40, and 30 km/h, and the design speeds of branch roads are 40, 30, and 20 km/h, where 40 km/h is taken as the default value.
$V_{m}/C_{m}$	0.15	Through investigation, the average daily motor vehicle saturation of roadside parking sections in cities is 0.15.
$V_{n}/C_{n}$	0.12	Through investigation, the average daily non-motor vehicle saturation of roadside parking sections in cities is 0.12.
$R_{b}$	0.38	Through investigation, the average spatial obstacle rate of roadside parking sections in cities is 0.38.
$R_{t}$	0.05	Through investigation, the average daily time obstacle rate of roadside parking sections in cities is 0.05.

## Data Availability

Not applicable.

## Appendix A

**Table A1:** Sample table of carbon compound contents in automobile exhaust gas.

Time	Carbon Dioxide	Carbon Monoxide	Hydrocarbon
	%	ppmC	ppmC
12:18:51	13.884	150	410
12:18:52	13.976	154.760303	402.4
12:18:53	13.99	172.26506	383.5
…	…	…	…

These data are obtained by measuring the carbon emissions in the exhaust gas generated during roadside parking. The experimental data show that carbon dioxide is the main carbon emission in the tail gas produced during vehicle parking. Please note that the ellipsis indicates that only part of the data is shown in the table. It means the same in other tables.

**Table A2:** BPR model parameters during roadside parking in scenario A (example).

Number	$V_{m}/C_{m}$	$V_{n}/C_{n}$	$v_{0}\;(\mathrm{km}/h)$	$R_{b}$	$R_{t}$	v (km/h)
1	0.046	0.323	40	0.32	0.029	44.737
2	0.092	0.1	40	0.32	0.029	46.282
3	0.123	0.426	40	0.32	0.029	43.323
4	0.138	0.15	40	0.32	0	37.193
5	0.154	0.2	40	0.32	0.029	43.007
6	0.154	0.2	40	0.32	0.029	42.82
7	0.154	0.15	40	0.32	0.029	40.329
8	0.154	0.1	40	0.32	0	42.104
9	0.169	0.15	40	0.32	0.029	38.174
10	0.169	0.1	40	0.32	0	42.489
…	…	…				
164	0.554	0.545	40	0.32	0.198	4.204
165	0.554	0.6	40	0.32	0.198	7.302
166	0.554	0.65	40	0.32	0.224	5.038
167	0.585	0.579	40	0.32	0.224	4.604
168	0.585	0.825	40	0.32	0.224	2.618
169	0.585	0.623	40	0.32	0.26	3.961
170	0.585	0.75	40	0.32	0.26	3.507
171	0.585	0.55	40	0.32	0.275	3.796
172	0.585	0.75	40	0.32	0.304	5.044
173	0.6	0.825	40	0.32	0.389	2.443

Please note that the ellipsis indicates that only part of the data is shown in the table. It means the same in other tables.

**Table A3:** BPR model parameters during roadside parking in scenario B1 (example).

Number	$V_{m}/C_{m}$	$V_{n}/C_{n}$	$v_{0}\;(\mathrm{km}/h)$	$R_{b}$	$R_{t}$	v (km/h)
1	0.092	0.15	40	0.63	0	40.677
2	0.169	0.375	40	0.63	0.039	38.136
3	0.169	0.3	40	0.63	0	37.766
4	0.169	0.325	40	0.63	0	37.669
5	0.169	0.325	40	0.63	0	35.314
6	0.169	0.288	40	0.63	0.039	32.758
7	0.169	0.35	40	0.63	0.039	31.281
8	0.169	0.425	40	0.63	0.039	37.199
9	0.185	0.3	40	0.63	0	35.792
10	0.185	0.325	40	0.63	0.039	35.317
…	…	…				
111	0.554	0.602	40	0.63	0.309	3.51
112	0.569	0.602	40	0.63	0.337	4.451
113	0.615	0.8	40	0.63	0.347	4.072
114	0.615	0.65	40	0.63	0.357	4.5
115	0.615	0.875	40	0.63	0.396	3.959
116	0.615	0.575	40	0.63	0	4.989
117	0.615	0.68	40	0.63	0	3.727
118	0.646	0.875	40	0.63	0	3.84
119	0.677	0.575	40	0.63	0.415	4.422
120	0.708	0.7	40	0.63	0.415	5.011

Please note that the ellipsis indicates that only part of the data is shown in the table. It means the same in other tables.

**Table A4:** BPR model parameters during roadside parking in scenario B2 (example).

Number	$V_{m}/C_{m}$	$V_{n}/C_{n}$	$v_{0}\;(\mathrm{km}/h)$	$R_{b}$	$R_{t}$	v (km/h)
1	0.123	0.2	40	0.25	0	47.085
2	0.154	0.45	40	0.25	0	46.961
3	0.2	0.274	40	0.25	0	46.458
4	0.2	0.281	40	0.25	0	44.833
5	0.215	0.2	40	0.25	0	24.92
6	0.215	0.225	40	0.25	0	39.367
7	0.231	0.225	40	0.25	0	36.424
8	0.231	0.333	40	0.25	0	44.344
9	0.246	0.25	40	0.25	0	40.499
10	0.246	0.3	40	0.25	0	39.437
…	…	…				
99	0.508	0.75	40	0.25	0.237	15.359
100	0.508	0.6	40	0.25	0.253	13.502
101	0.554	0.6	40	0.25	0.266	10.608
102	0.554	0.6	40	0.25	0.282	5.633
103	0.6	0.618	40	0.25	0.315	4.676
104	0.677	0.7	40	0.25	0.327	2.648
105	0.677	0.6	40	0.25	0.327	4.953
106	0.677	0.583	40	0.25	0.339	3.62
107	0.738	0.667	40	0.25	0.372	4.654
108	0.808	0.75	40	0.25	0.458	2.892

Please note that the ellipsis indicates that only part of the data is shown in the table. It means the same in other tables.

**Table A5:** Relationship between carbon emissions and velocity (example).

Number	v (km/h)	E (g)
1	0	0.923
2	0.096	0.9
3	2.01	0.876
4	3.003	0.852
5	4.333	0.828
6	5.625	0.807
7	6.14	0.788
8	7	0.76
9	8.11	0.738
10	8.665	0.716
…	…	…
74	41	0.236
75	42.777	0.228
76	43	0.219
77	44	0.212
78	44.65	0.205
79	46	0.198
80	47.098	0.192
81	48	0.187
82	48.969	0.18
83	50	0.175

Please note that the ellipsis indicates that only part of the data is shown in the table. It means the same in other tables.
